# Case Report: *Plasmodium vivax* Sporozoite Melanization in the Midgut and Salivary Gland of the Malaria Vector *Anopheles darlingi*

**DOI:** 10.4269/ajtmh.23-0349

**Published:** 2024-02-13

**Authors:** Najara Akira Costa dos Santos, Alessandra da Silva Bastos, Jéssica Evangelista Araújo, José Daniel Costa Pontual, Jansen Fernandes Medeiros, Joseph Michael Vinetz, Maisa da Silva Araujo

**Affiliations:** ^1^ Plataforma de Produção e Infecção de Vetores da Malária- PIVEM, Laboratório de Entomologia, Fiocruz Rondônia, Porto Velho, Rondônia, Brazil;; ^2^ Instituto Nacional de Epidemiologia da Amazônia Ocidental (INCT-EpiAMO), Porto Velho, RO, Brazil;; ^3^ Programa de Pós-graduação em Biologia Experimental – Universidade Federal de Rondônia/Fiocruz Rondônia, Porto Velho, Rondônia, Brazil;; ^4^ Section of Infectious Diseases, Department of Internal Medicine, Yale School of Medicine, New Haven, Connecticut, United States of America;; ^5^ Programa de Pós-Graduação em Conservação e uso de Recursos Naturais – PPGReN, Fundação Universidade Federal de Rondônia, Porto Velho, Rondônia, Brazil;; ^6^ Laboratório de Pesquisa Translacional e Clínica, Centro de Pesquisa em Medicina Tropical, Porto Velho, Rondônia, Brazil

## Abstract

*Anopheles darlingi* is the primary malaria vector in the Amazon region and is highly susceptible to both *Plasmodium vivax* and *Plasmodium falciparum* parasites. Although anopheline mosquitoes may develop melanotic encapsulation in response to *Plasmodium* parasites, there is no record of *An. darlingi* exhibiting a melanization response to *P. vivax*, the main malaria parasite in the Americas. Here, we report the occurrence of *P. vivax* sporozoite melanization in *An. darlingi* mosquitoes.

## INTRODUCTION

Malaria remains a significant infectious disease, responsible for approximately 250 million cases worldwide each year. In the Americas, *Plasmodium vivax* is the primary pathogenic, accounting for more than 70% of malaria cases,^1^ and *Anopheles darlingi* is the principal malaria vector in the Amazon region. Anopheline mosquitoes develop various immune responses against *Plasmodium* and other pathogens,^2^ including melanization, a complex humoral innate immunity response related to wound healing and pathogen encapsulation.^3^ Here, for the first time, we report cases of *P. vivax* sporozoite melanization in *An. darlingi* and investigate whether a higher intensity of infection improves melanization responses against sporozoites in *An. darlingi*.

## CASES

**Figure 1.**
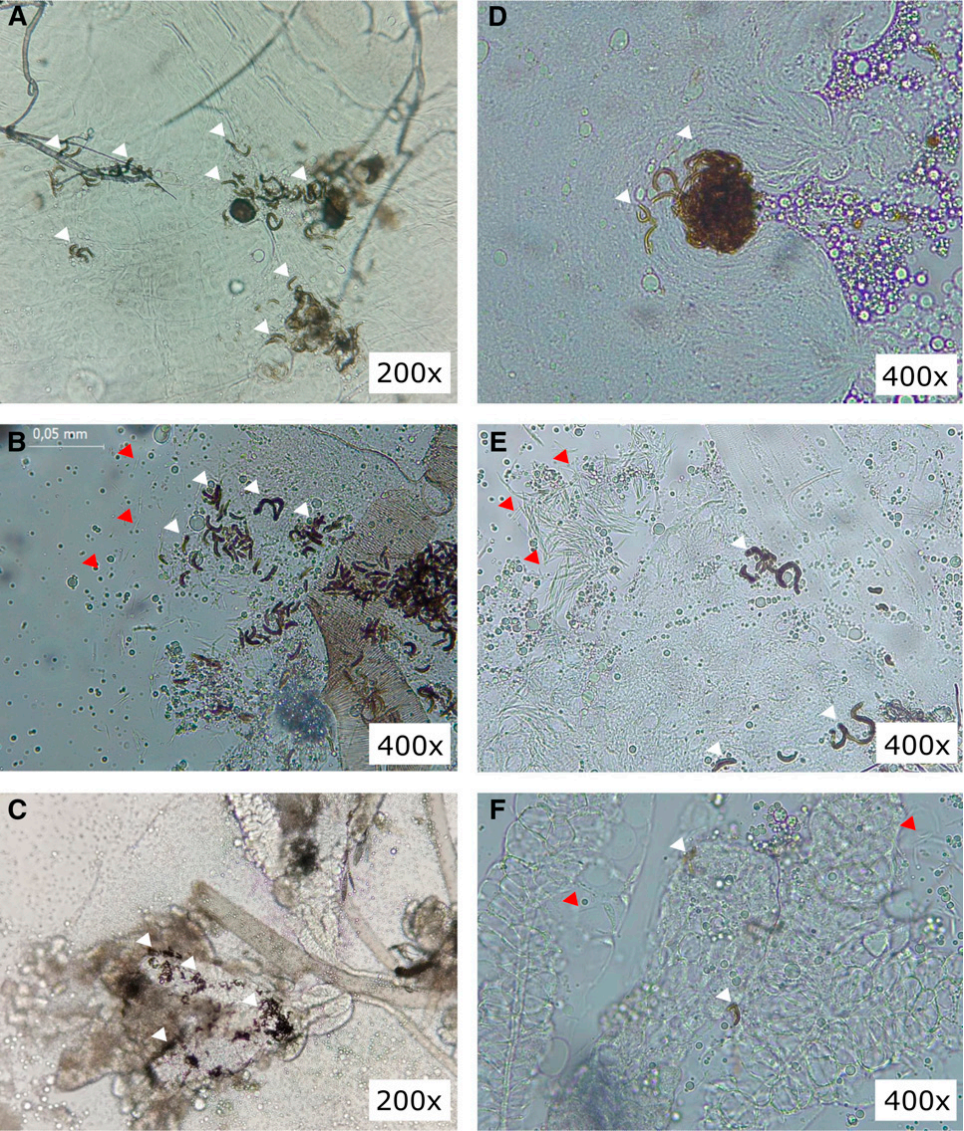
Melanized *Plasmodium vivax* sporozoite in *Anopheles darlingi* mosquitoes. (**A** and **D**) Melanized sporozoites outside the midgut. (**B**, **C**, **E**, and **F**) Melanized and nonmelanized sporozoites outside the salivary glands. White arrowheads indicate melanized sporozoites, and red arrowheads indicate free sporozoites. The white box indicates the total magnification under light microscopy. All images were obtained at 14 days post infection.

Melanized sporozoites were observed in individuals of the *An. darlingi* species during the routine dissection of salivary glands (SGs) for sporozoite purification at 14 days post infection (dpi). Records of melanized sporozoites were found in bunches (Figure 1A–C) or individually (Figure 1E and F), both outside the SGs and outside the midgut (MG) of infected mosquitoes (Figure 1D). These mosquitoes were obtained from a Brazilian *An. darlingi* colony that has been maintained under laboratory rearing conditions since 2018^4^ and were fed on blood samples from *P. vivax*–positive patient donors by direct membrane feeding assay (DMFA).

After randomly observing melanized sporozoite in our mosquitoes, we wondered whether the melanization response could be higher in mosquitoes with a greater *P. vivax* infection intensity. Therefore, we performed three independent experimental infections to assess the melanization response of *An. darlingi* mosquitoes against the *P. vivax* sporozoite stage under two infection conditions. Mosquitoes obtained from the *An. darlingi* colony were deprived of sugar feeding for up to 12 hours before the blood meal. Two batches of 50 female *An. darlingi* mosquitoes, aged 3 to 5 days, were separated. For the first group, plasma from a *P. vivax*–positive blood sample was replaced with inactivated AB^+^ serum in a 1:1 (erythrocytes-to-serum) ratio, and this mixture was offered to the mosquitoes by DMFA. This group was designated SR. The second group was fed on the same *P. vivax*–positive blood sample but without serum replacement and was referred to as WB. The serum replacement approach is commonly used to increase infection rates in anopheline mosquitoes.^5^

After the blood meal, only completely engorged female mosquitoes were maintained in the study. At 14 dpi, the MGs and SGs of approximately 20 females from each group were dissected under a stereo microscope. Midguts were stained with 0.2% mercurochrome. Melanized sporozoites in both organs were visualized and counted using a microscope (×100).

The experiments were conducted with the approval of the Ethics Committee at the Centro de Pesquisa em Medicina Tropical (protocol number 530,106). All participants invited to participate in the study met the following criteria: positive diagnosis for *P. vivax* malaria, age ≥ 18 years, not pregnant, not indigenous, absence of severe or complicated malaria, and providing oral and signed consent to participate in the study. Oocyst intensity between the WB and SR groups, as well as the number of melanized sporozoites between WB-SG versus SR-SG and WB-MG versus SR-MG were compared using the Mann-Whitney test. The number of melanized sporozoites between WB-SG versus WB-MG and SR-SG versus SR-MG were compared using the Wilcoxon test for paired samples. Finally, the prevalence of mosquitoes with melanized sporozoites between the groups were compared by Fisher’s exact test. The 95% CIs were calculated by the Wald method, and Bonferroni correction was applied to the *P*-values.

**Figure 2.**
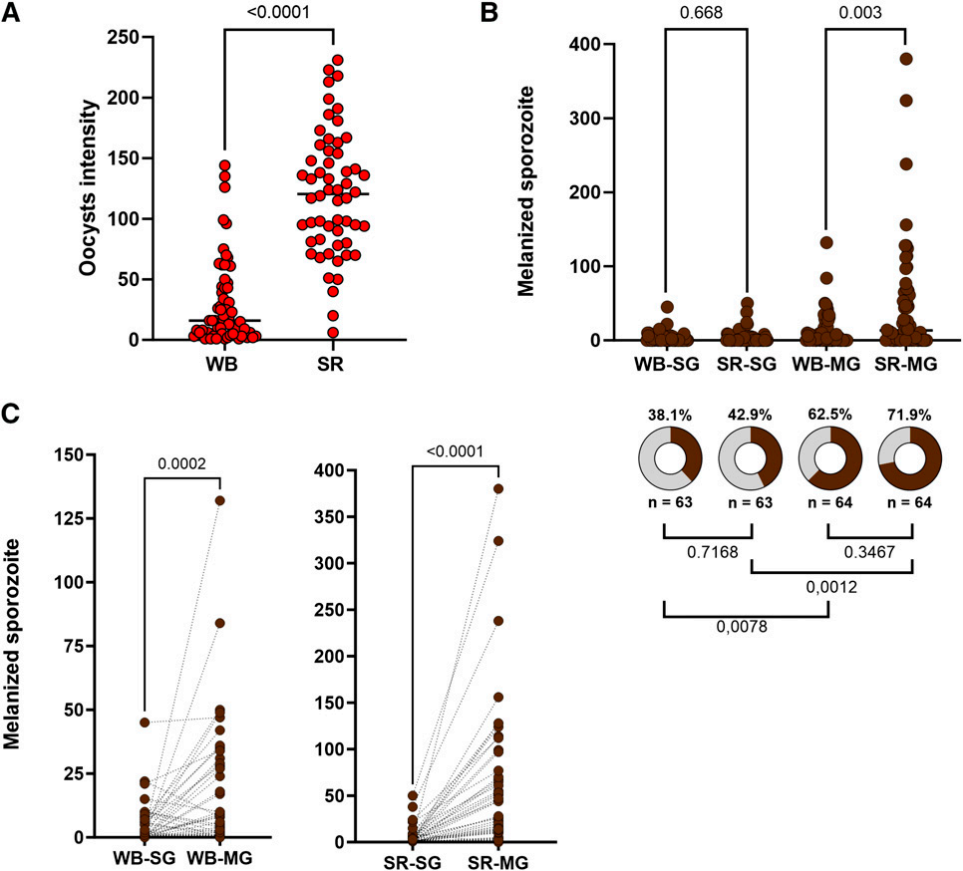
Sporozoite melanization in *Anopheles darlingi* infected with *Plasmodium vivax*. (**A**) Oocyst intensity of mosquitoes fed under whole blood (WB) or serum replacement (SR) conditions. Each red circle represents a mosquito dissected. (**B**) Number of melanized sporozoites in each experimental group: whole blood–salivary gland (WB-SG); serum replacement–salivary gland (SR-SG); whole blood–midgut (WB-MG), and serum replacement–midgut (SR-MG) and the prevalence of mosquitoes with at least one melanized sporozoite. (**C**) Salivary gland and midgut paired melanized sporozoite in the WB group and the SR group. For (**B)** and (**C)** plots, each brown circle represents a salivary gland or a midgut dissected. The gray color in the pizza plot represents the proportion of mosquitoes without melanized sporozoites and the brown color represents the proportion of mosquitos with melanized sporozoites.

Serum replacement before DMFA showed a significant increase in oocyst intensity (WB oocyst median = 16; SR oocyst median = 120.5; Mann-Whitney test, *U* = 246.5; *P* <0.0001) (Figure 2A). The number of melanized sporozoites was statistically higher in the MGs of the SR group than in the MGs of the WB group (WB melanized sporozoite median = 2; SR melanized sporozoite median = 13.5; Mann-Whitney test, *U* = 1,449; *P* = 0.0034). However, the quantity of melanized sporozoites between the SGs of the WB and SR groups did not show a statistical difference (WB melanized sporozoite median = 0; SR melanized sporozoite median = 0; Mann-Whitney test, *U* = 1,906; *P* = 0.6682). The proportion of mosquitoes with melanized sporozoites in the SGs was 38.1% (95% CI: 27.1–50.5) in the WB group and 42.9% (95% CI: 31.4–55.1) in the SR group. For MGs, 62.5% of mosquitoes had melanized sporozoites (95% CI: 50.2–73.3) in the WB group and 71.9% (95% CI: 59.8–81.5) in the SR group. However, no significant difference was observed between WB-SG versus SR-SG, as shown in Figure 2B.

When the MGs and SGs of the same group were compared, we observed more melanized sporozoites were present in the MGs than in the SGs, regardless of the group, whether WB or SR (Figure 2C) (WB group: Wilcoxon paired test, *W* = 585; *P* = 0.0002; SR group: Wilcoxon paired test, *W* = 1,120; *P* < 0.0001). In addition, a higher proportion of mosquitoes exhibited melanized sporozoites in the MGs than in the SGs for both experimental groups, WB and SR (Figure 2B).

## DISCUSSION

Here, we report our observations of melanized *P. vivax* sporozoites located outside the MGs and SGs of *An. darlingi* on day 14 after the ingestion of a blood meal from *P. vivax*–infected human donors. Although melanization is a primary immune system response in insects, it is considered rare in susceptible strains.^6^^,^^7^ In refractory anopheline species, melanization is described primarily against the ookinete stages, as observed in the stable line *Anopheles gambiae* L3-5 used in immune response studies in anopheline mosquitoes.^8^^–^^10^

*Anopheles darlingi* is a highly susceptible neotropical anopheline species to *P. falciparum* and *P. vivax* malaria parasites^11^^,^^12^; however, it has been shown to have a lower sporozoite load than African and Asian anophelines.^13^^–^^15^ To date, there have been no records of melanization in *An. darlingi.* For *P. vivax* and neotropical anopheline models, there are only two reports available in the literature about melanization. In 2000, Moreno and Berti^16^ studied the rate of oocyst melanization in an Anopheles *aquasalis* field captured in Venezuela and found melanized sporozoites of *P. vivax* in the hemocoel. In the second study, Hernández-Martínez et al.^17^ assessed the cellular-mediated reaction of *Anopheles albimanus*, a neotropical anopheline known to have poor vectorial competence to *P. vivax,* by injecting sporozoites through the mosquito thorax and observed the melanization of some of these parasites.

Previously, Simões et al.^18^ suggested that melanization in anopheline mosquitoes is directly dependent on the infection intensities of *Plasmodium* infection, as demonstrated in the *An. albimanus–Plasmodium berghei* and *P. falciparum* models. In this study, we induced a high *P. vivax* infection in *An. darlingi* by replacing plasma blood with inactivated serum AB^+^. As a result, we found more melanized sporozoites in the MGs of highly infected mosquitoes, as expected. Interestingly, we noted a higher number of melanized sporozoites outside MGs than outside SGs, regardless of the infection load.

Owing to some technique limitations, we were unable to measure the proportion of melanized sporozoites in the mosquitoes. However, nonmelanized sporozoites were present in all SGs where melanized sporozoites were observed. This suggests that the immune response of *An. darlingi* mosquitoes may be effectively evaded by *P. vivax*, as observed in the literature.^19^ The success of evasion seems to be genetically determined and may be dependent on *Plasmodium*–vector compatibility, as shown for the surface protein Pfs47, which allows *P. falciparum* to survive in their sympatric vectors.^20^ Another surface protein implicated in the evasion of mosquito immune response is the circumsporozoite protein (CSP). Studies have shown that conformational changes or posttranslational modification (glutaminyl cyclase) of this surface protein prevents the melanization of oocysts and sporozoites of *Plasmodium*.^21^^,^^22^ In the case of *P. vivax*, although it may evade the immunological response of *An*. *darlingi*, the vector’s ability to melanize free sporozoites in the hemocoel could be important in decreasing the transmission success of *P. vivax*.

In addition to genetic factors,^9^^,^^19^ nongenetic factors such as environmental conditions and the developmental stage of mosquitoes can influence them to mount a melanization response. For example, age and reproductive status,^6^^,^^23^ larval rearing conditions,^24^ and dietary resources in the adult stage^6^^,^^25^ can affect their response. Therefore, given the importance of *P. vivax* in malaria transmission in the Americas, particularly in the Amazon region, studying the ability of *An. darlingi* to develop a melanization response under different conditions, investigating whether field-captured *An. darlingi* exhibits a melanization response to *P. vivax*, and understanding the immune evasion strategies used by *P. vivax* may offer valuable insights for studies aimed at blocking malaria transmission.
